# Supplementary material for: Site-specific effects of neurosteroids on GABAA receptor activation and desensitization
Source: eLife. 2020 Sep 21;9:e55331. doi: 10.7554/eLife.55331 (PMC7532004; doi:10.7554/eLife.55331)
Supplement: Figure 7—source data 1. — Kd and Bmax for [3H]muscimol binding isotherms in the α1β3 GABAARs WT and mutated receptors. Kd and Bmax values are compared using one-way ANOVA with Bonferroni's multiple comparisons test. Data are presented as mean ± SEM (n = 6 for WT; n = 3 for mutated receptors). [file elife-55331-fig7-data1.docx]

|  | **K_d_ (nM)** | **B_max_ (pmol/mg)** |
| --- | --- | --- |
| **WTα_1_β_3_** | 48.1 ± 4.0 | 12.1 ± 1.4 |
| **α_1_(Q242L)β_3_** | 44.3 ± 4.6 (*P* > 0.99 vs. WT) | 9.9 ± 0.6 (*P* > 0.99 vs. WT) |
| **α_1_(N408A/Y411F)β_3_** | 60.7 ± 7.5 (*P* = 0.89 vs. WT) | 0.9 ± 0.1 (**P* < 0.01 vs. WT) |
| **α_1_(V227W)β_3_** | 54.1 ± 5.8 (*P* > 0.99 vs. WT) | 11.6 ± 0.4 (*P* > 0.99 vs. WT) |
| **α_1_β_3_(Y284F)** | 46.6 ± 3.8 (*P* > 0.99 vs. WT) | 10.6 ± 0.9 (*P* > 0.99 vs. WT) |
