# Supplementary material for: Site-specific effects of neurosteroids on GABAA receptor activation and desensitization
Source: eLife. 2020 Sep 21;9:e55331. doi: 10.7554/eLife.55331 (PMC7532004; doi:10.7554/eLife.55331)
Supplement: Figure 7—source data 2. — Kd and Bmax for the [3H]muscimol binding isotherms in Figure 7C–D. Kd values are compared using unpaired t-test. Statistical differences between the whole curves are analyzed using two-way ANOVA. Data are presented as mean ± SEM (n = 3). [file elife-55331-fig7-data2.docx]

| **WTα_1_β_3_** | **K_d_ (nM)** | | **B_max_ (pmol/mg)** |
| --- | --- | --- | --- |
| Control | 35.5 ± 4.2 | | 9.8 ± 0.8 |
| Etomidate (100 μM) | 19.9 ± 1.6 (**P* = 0.03 vs. control) | | 10.1 ± 0.5 |
| 3α5αP (30 μM) | 12.6 ± 1.3 (**P* < 0.01 vs. control) | | 10.3 ± 0.7 |
| KK148 (30 μM) | 15.1 ± 1.6 (**P* = 0.01 vs. control) | | 10.8 ± 0.9 |
| **α_1_(N408A/Y411F)β_3_** |  | |  |
| Control | 68.2 ± 12.3 (*P* = 0.07 vs. WT) | | 0.90 ± 0.18 |
| Etomidate (100 μM) | 27.0 ± 3.9 (**P* = 0.03 vs. control) | | 0.97 ± 0.23 |
| 3α5αP (30 μM) | 98.4 ± 29.6 (*P* = 0.40 vs. control) | | 0.93 ± 0.16 |
| KK148 (30 μM) | 72.8 ± 12.8 (*P* = 0.81 vs. control) | | 0.85 ± 0.22 |
| **WTα_1_β_3_** | | **Statistical difference between the curves** | |
| Control vs. Etomidate | | *F*_7,32_ = 3.27, **P* < 0.01 | |
| Control vs. 3α5αP | | *F*_7,32_ = 4.27, **P* < 0.01 | |
| Control vs. KK148 | | *F*_7,32_ = 5.96, **P* < 0.01 | |
| **α_1_(N408A/Y411F)β_3_** | |  | |
| Control vs. Etomidate | | *F*_7,32_ = 2.21, *P* = 0.06 | |
| Control vs. 3α5αP | | *F*_7,32_ = 0.71, *P* = 0.66 | |
| Control vs. KK148 | | *F*_7,32_ = 0.29, *P* = 0.95 | |
