# Supplementary material for: Site-specific effects of neurosteroids on GABAA receptor activation and desensitization
Source: eLife. 2020 Sep 21;9:e55331. doi: 10.7554/eLife.55331 (PMC7532004; doi:10.7554/eLife.55331)
Supplement: Figure 11—source data 1. [file elife-55331-fig11-data1.docx]

**Vina docking scores for the β_3_(+)–α_1_(-) intersubunit site**

|  | Model Vina score | 6I53 Vina score |
| --- | --- | --- |
| 3α5αP | -8.0 | -8.9 |
| 3β5αP | -7.9 | -8.7 |

**Vina docking scores for the α_1_ intrasubunit site**

|  | Model Vina score | 6I53 Vina score |
| --- | --- | --- |
| 3α5αP | -7.2 | -6.5 |
| 3β5αP | -7.4 | -6.1 |

**Vina docking scores for the β_3_ intrasubunit site**

|  | Model Vina score | 6I53 Vina score |
| --- | --- | --- |
| 3α5αP | -5.7 | -5.2 |
| 3β5αP | -5.5 | -4.6 |
